# Sensorimotor Challenges in Minimally Invasive Surgery: A Theoretically-Oriented Review

**DOI:** 10.1177/00187208241263684

**Published:** 2024-07-22

**Authors:** Christopher L. Hewitson, Matthew J. Crossley, John Cartmill, David M. Kaplan

**Affiliations:** 1 5755 Yale University, USA; 2 7788 Macquarie University, Australia

**Keywords:** medical simulation/training and assessment, simulation and training, skilled, performance, motor learning, motor control, surgical training, sensorimotor-adaptation

## Abstract

**Objective:**

This review surveys the literature on sensorimotor challenges impacting performance in laparoscopic minimally invasive surgery (MIS).

**Background:**

Despite its well-known benefits for patients, achieving proficiency in MIS can be challenging for surgeons due to many factors including altered visual perspectives and fulcrum effects in instrument handling. Research on these and other sensorimotor challenges has been hindered by imprecise terminology and the lack of a unified theoretical framework to guide research questions in the field.

**Method:**

We conducted a systematic survey of the MIS literature, focusing on studies investigating sensorimotor challenges affecting laparoscopic performance. To provide a common foundation for cross-study comparisons, we propose a standardized taxonomy that distinguishes between different experimental paradigms used in the literature. We then show how the computational motor learning perspective provides a unifying theoretical framework for the field that can facilitate progress and motivate future research along clearer, hypothesis-driven lines.

**Results:**

The survey identified diverse sensorimotor perturbations in MIS, which can be effectively categorized according to our proposed taxonomy. Studies investigating monitor-, camera-, and tool-based perturbations were systematically analyzed, elucidating their impact on surgical performance. We also show how the computational motor learning perspective provides deeper insights and potential strategies to mitigate challenges.

**Conclusion:**

Sensorimotor challenges significantly impact MIS, necessitating a systematic, empirically informed approach. Our proposed taxonomy and theoretical framework shed light on the complexities involved, paving the way for more structured research and targeted training approaches to enhance surgical proficiency.

**Application:**

Understanding the sensorimotor challenges inherent to MIS can guide the design of improved training curricula and inform the configuration of setups in the operating room to enhance surgeon performance and ultimately patient outcomes. This review offers key insights for surgeons, educators, and researchers in surgical performance and technology development.

## Introduction

Laparoscopic minimally invasive surgery (MIS) has revolutionized surgical medicine in recent decades (Darzi & Munz, 2004), leading to shorter recovery times, lower infection rates, and reductions in a range of other postoperative complications compared to open surgery (Braga et al., 2002; Cuschieri, 1995; Nguyen et al., 2007; Subramonian et al., 2004; Varela et al., 2010). Nevertheless, achieving and maintaining proficiency in MIS can be challenging (Bennett et al., 1997; Birkmeyer et al., 2013; Braga et al., 2002; Cagir et al., 1994; Grantcharov & Funch-Jensen, 2009; Hamdorf & Hall, 2000; Vickers et al., 2009), and intraoperative complication rates can sometimes exceed that of open surgery (Club, 1991; Deziel et al., 1993). Additionally, there is growing evidence that laparoscopic training is associated with increased mental workload and stress compared to training in open or robotic-assisted minimally invasive surgical settings (Klein et al., 2008, 2012; Zheng et al., 2010).

**Figure 1.**
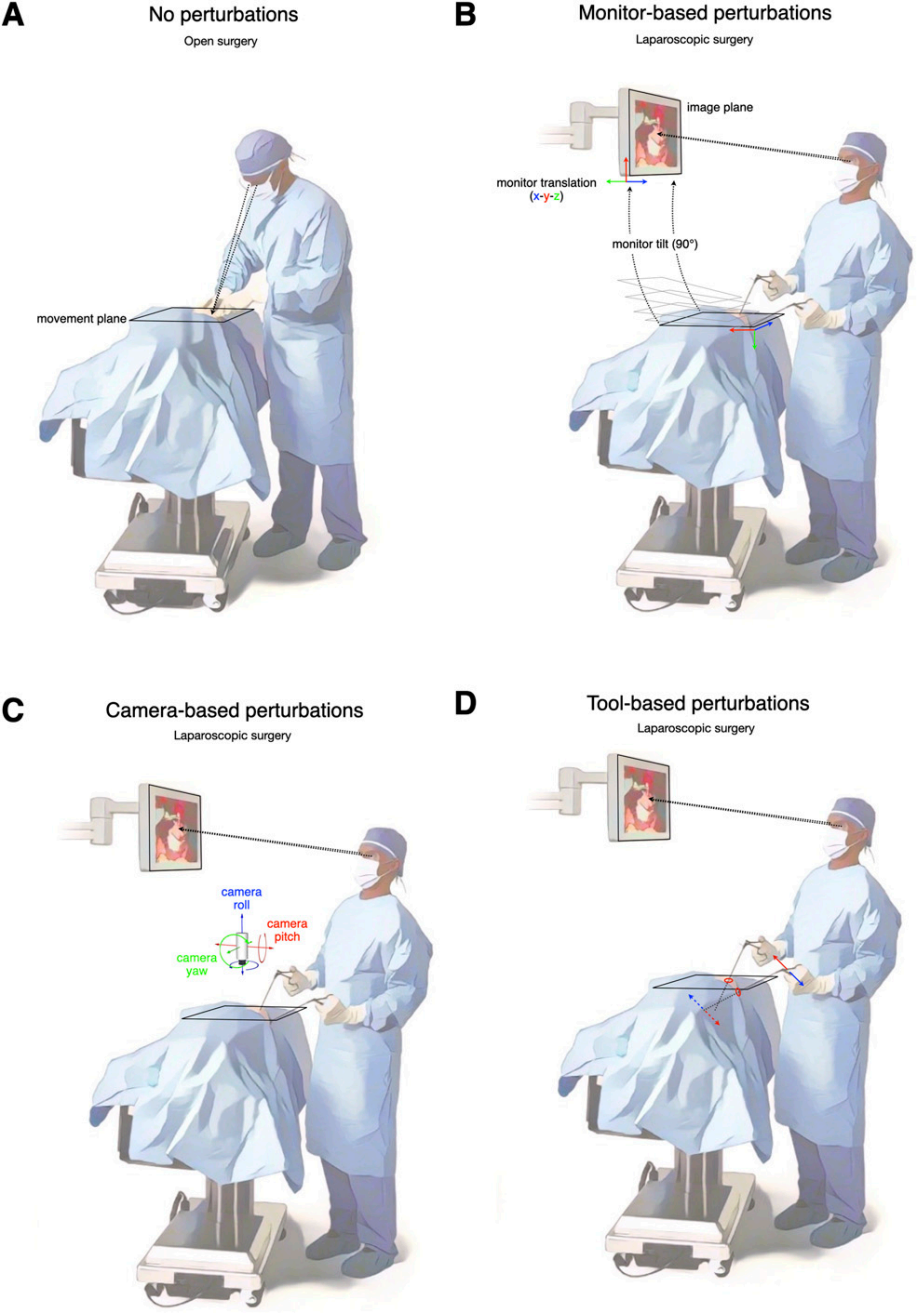
Open surgery compared to the different types of sensorimotor perturbations experienced in MIS. (a) In open surgery, the native reference frame of vision and the physical workspace are closely, if not perfectly, aligned. (b) In laparoscopic surgery, monitor-based sensorimotor perturbations arise from translational and rotational displacements of the monitor (where visual feedback is provided) relative to the physical workspace. (c) Camera-based perturbations arise from displacements of the laparoscopic camera (roll, pitch, yaw) relative to the physical workspace, especially the movement plane. (d) Tool-based perturbations arise from the complex motion of laparoscopic instruments or tools such as when hand motion is inverted relative to instrument tip motion. Solid arrows indicate hand movement directions and dashed arrows indicate instrument tip movement directions. Colors indicate paired instrument tip movements. Only the surgeon is shown. However, the camera operator faces similar challenges, which in turn compound the challenges facing the surgeon.

Various factors contribute to the difficulties encountered in MIS (Ballantyne, 2002; Cuschieri, 1995), including degraded haptic feedback (Westebring-van der Putten et al., 2008) and lack of depth perception (Beattie et al., 2021; Hofmeister et al., 2001; Sakata et al., 2016). However, sensorimotor challenges likely have an outsized impact (Breedveld et al., 1999; Breedveld & Wentink, 2001; Crossley et al., 2021; Stassen et al., 2001; Wentink, 2001). Unlike open surgery, MIS involves indirect interaction with the operative field mediated by cameras, monitors, and complex tools (compare Figure 1(a) vs. Figure 1(b)–(d)). Surgeons rely on two-dimensional (2D) or three-dimensional (3D) representations of the scene displayed on monitors, rather than directly looking at their hands and instruments (compare Figure 1(a) vs. Figure 1(b)–(d)). And this view is usually provided by the less experienced surgeon or trainee controlling the laparoscopic camera with its own confounding optics and degrees of freedom. The fact that the image is controlled by another human, often less experienced, means that the surgeon is rarely looking at the operative site at the exact orientation, angle and distance they would prefer.

The nature of laparoscopic instruments poses additional challenges for sensorimotor control. In MIS, hand and instrument tip often move in opposite directions. This complication is known as the *fulcrum effect* (Gallagher et al., 1998). Additionally, the lever action of laparoscopic instruments causes straight-hand trajectories to produce curved trajectories at the distal end of the tool (Heuer & Sülzenbrück, 2009). Finally, the position of the instrument’s pivot point affects its gain, altering the relationship between hand and instrument movements (Emam et al., 2000).

These characteristics of MIS have practical consequences. First, there is a vast range of potential modifications and configurations that laparoscopic surgeons can explore to optimize their performance. Currently, lacking a solid knowledge base, surgeons rely on personal preferences and experiences. Second, changes in the position and orientation of monitors, cameras, and surgical instruments can disrupt the typical relationship between the surgeon’s movements and sensory feedback (see Figure 1), significantly impacting performance. Such changes, known as sensorimotor perturbations, induce initial performance errors that necessitate motor adaptation to refine future movements (Krakauer et al., 2019; Smith et al., 2010; Wolpert et al., 2011). Daily motor learning involves adapting movements to compensate for various sensorimotor perturbations, such as environmental or bodily changes, manipulating new objects or tools, and refining existing motor skills.

Consider a simple example. The outcome of a golf swing depends on both intrinsic properties of the player’s swing and fluctuations in the external environment (e.g., wind direction, club selection, and flex characteristics of the shaft). Skilled golfers must compensate for these and other environmental variables to consistently hit accurate shots. Similarly, expert surgeons must adapt their movements to maintain stable performance in response to changes in the MIS environment.

Over the past few decades, a growing number of studies have sought to investigate the sensorimotor factors that make MIS challenging. Using a diverse range of validated laparoscopic simulator tasks and performance metrics (Boza et al., 2017; Dawe et al., 2014; Fried et al., 1999; Sinitsky et al., 2012; Stefanidis et al., 2012), investigators have made considerable progress linking changes in task performance to a host of environmental manipulations. Despite this expanding experimental literature, our understanding remains fragmented, and there is little consensus on how to optimize the surgical environment or mitigate sensorimotor perturbations during MIS procedures. One reason for this lack of progress is the imprecise terminology used in experimental studies, hindering meaningful comparisons and replication. Furthermore, the field lacks a unifying theoretical framework to guide research questions and make experimental design choices more systematic.

To address these issues and accelerate progress, we propose a (1) standardized terminology for describing experimental manipulations used in the field and (2) the integration of a theoretical framework from computational motor control and motor learning (Franklin & Wolpert, 2011; McNamee & Wolpert, 2019; Shadmehr & Wise, 2005; Wolpert et al., 2001, 2011; Wolpert & Flanagan, 2001). The science of motor learning offers valuable insights into how humans adapt movements to changes in the environment and refine skills, which can inform the MIS literature. This perspective complements the ergonomic viewpoint dominant in the field (Berguer et al., 1999; Gabrielson et al., 2021). This review provides the first comprehensive survey of the literature on sensorimotor challenges in MIS in several decades (Breedveld & Wentink, 2001) and applies a motor learning perspective to reveal some of the deeper structure in the MIS literature (see Jarc & Nisky, 2020 for a similar approach applied to robotic-assisted MIS). Our aim is to facilitate the generation of clear, testable predictions based on previous research, promoting reproducible and progressive science (Ioannidis, 2005; Lakatos, 1970).

## Review Process

**Figure 2.**
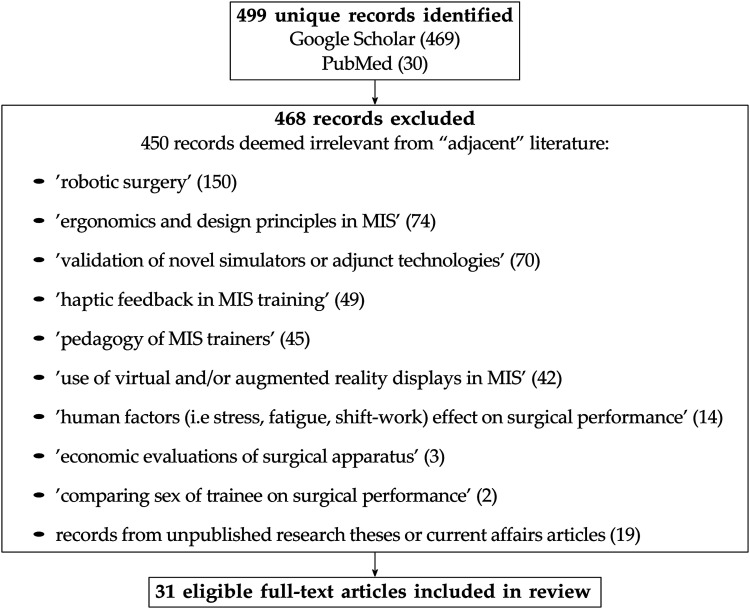
Flow of review process.

Given the diverse range of paradigms, conditions, and metrics employed in the reviewed studies, a narrative review was deemed more appropriate than a systematic review (Baumeister, 2013; Siddaway et al., 2019). Nonetheless, we adhered to key PRISMA guidelines for study identification, screening, and inclusion (Page et al., 2021). Relevant studies were identified by entering the following keyword search terms into Google Scholar and PubMed databases: “minimally invasive surgery,” “laparoscopy surgery,” “motor,” “skill,” “learning,” “endoscope,” “monitor,” “image,” “tool,” “rotation,” “alignment”. In total, 499 unique records were identified through Google Scholar (*n* = 469) and PubMed (*n* = 30). Each unique record was subsequently screened and excluded if it was (a) deemed irrelevant following a detailed content analysis of the associated article (*n* = 449) or (b) because it was either an unpublished research thesis or a current affairs article (*n* = 19). The remaining 31 full-text articles were included in the review (Figure 2).

## Towards a Standardized Taxonomy of MIS Paradigms

To precisely categorize the sensorimotor perturbations in minimally invasive surgery (MIS) described in the experimental literature, we adopt the concept of a reference frame from physics, which defines an object’s spatial position, orientation, and motion. An object can move independently along six degrees of freedom (DoFs): three translational DoFs along the x, y, and *z* axes (forward/backward, left/right, up/down; depicted in Figure 1(b)) and three rotational DoFs around the x, y, and *z* axes (roll, pitch, yaw; depicted in Figure 1(c)) (Wolpert et al., 2001).

In open surgery, the surgeon’s visual workspace and physical workspace align closely. However, during MIS procedures, the laparoscopic camera and visual displays can move and rotate relative to the physical workspace. We can describe the camera, monitors, and surgeon as occupying different reference frames and characterize sensorimotor perturbations based on misalignments with respect to the reference frame anchored to the surgeon’s physical workspace. For simplicity, we focus on a primary plane of movement within the overall workspace, referred to as the movement plane (as depicted in Figure 1(a)–(d)).

Based on this framework, the MIS literature on the effects of sensorimotor perturbations can be categorized into three main types: monitor-based, camera-based, and tool-based perturbations.

Monitor-based sensorimotor perturbations occur due to the motion of the external video monitor relative to the physical workspace. In modern operating rooms, adjustable monitors are commonly used, allowing for spatial dissociation between the monitor location and the surgical workspace. For example, there may be vertical translation and rotation (pitch) differences between the movement plane and the monitor plane (as is depicted in Figure 1(b)).

**Figure 3.**
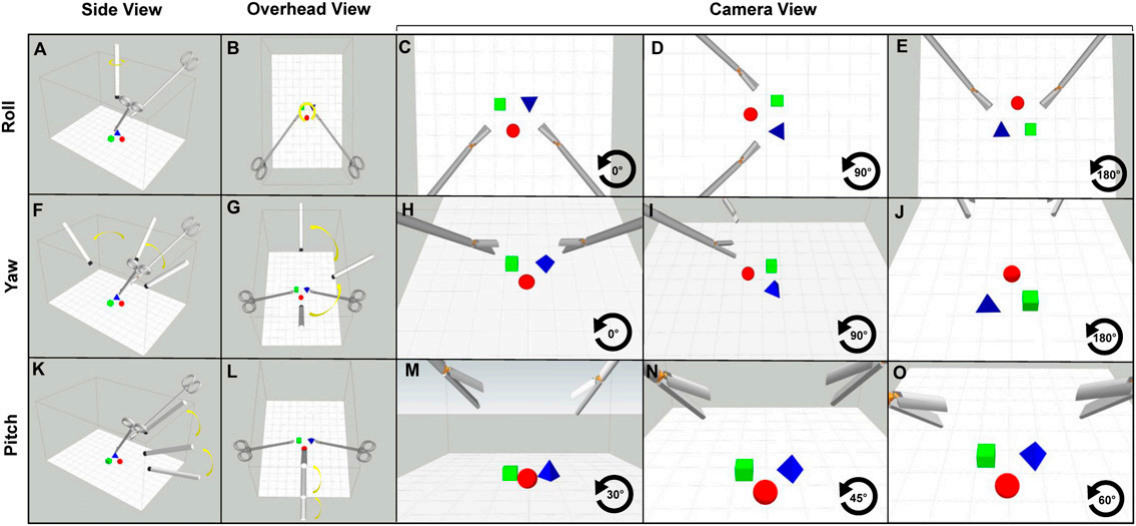
Different views of the MIS experimental workspace. (a–e) Workspace and camera positions for experimental paradigms probing the influence of camera roll angle changes on motor performance. Panels from left to right depict side view, overhead view, and camera views (0° roll, 90° roll, 180° roll), respectively. (f–j) Workspace and camera positions for experimental paradigms probing the influence of camera yaw angle changes on motor performance. Panels from left to right depict side view, overhead view, and camera views (0° yaw, 90° yaw, 180° yaw), respectively. (k–o) Workspace and camera positions for experimental paradigms probing the influence of camera pitch angle changes on motor performance. Panels from left to right depict side view, overhead view, and camera views (30° pitch, 45° pitch, 60° pitch), respectively.

Camera-based sensorimotor perturbations arise from the motion of the laparoscopic camera relative to the physical workspace (Figure 1(c)). Changes in camera roll, pitch, and yaw introduce rotations and/or translations of visual feedback relative to the workspace or movement plane (Figure 3).

Tool-based sensorimotor perturbations result from the complex motion of laparoscopic instruments or tools (Figure 1(d)). In MIS, long, thin instruments are inserted through small incisions (ports) and serve as pivot points (Breedveld et al., 1999; Gallagher et al., 1998; Gould & Frydman, 2007; Wentink et al., 2000). The motion of the instrument tip is often reversed relative to the hand motion at the handle, a phenomenon known as the *fulcrum effect* (Crothers et al., 1999; Gallagher et al., 1998). This inversion of the normal relationship between hand motion and visual feedback direction can pose challenges for inexperienced users. Surgeons may also encounter variations in force and motion scaling depending on the pivot point along the instrument shaft (Emam et al., 2000). Most laparoscopic instruments add an additional DoF by allowing the shaft to rotate relative to the handle so that the position of the jaw is independent of the surgeon’s hand position.

In the following sections, we employ this taxonomy to elucidate the current understanding and remaining gaps regarding the influence of sensorimotor perturbations on MIS performance in the literature (Table 1). To provide context, we also review relevant findings from the field of motor learning.

**Table 1.** Summary Table of MIS Literature.

Study	Cohort(s)	Stated independent variable(s)	Perturbation type(s)	Dependant variable(s)
Hanna et al., (1998)	Expert	“location of image display”	monitor-based (vertical, horizontal translation, pitch)	task completion time
Erfanian et al., (2003)	Expert	“video image position”	monitor-based (vertical translation, pitch)	task completion time
Matern et al., (2005)	naive	“monitor position”	monitor-based (vertical, horizontal translation, pitch)	task performance
Haveran et al., (2007)	naive, expert	“monitor position,” “camera position”	monitor-based (horizontal translation, pitch)	task completion time
			camera-based (yaw)	task completion time
Hernandez et al. (2014)	naive	“visual display location”	monitor-based (horizontal translation)	task completion time
				error rate
				path length
				motion smoothness.
Omar et al. (2005)	naive	“gaze-down display location”	monitor-based (pitch)	task completion time
				error rate
White et al., (2016)	naive	“monitor position”	monitor-based (horizontal translation, pitch)	task completion time
				movement accuracy movement smoothness
Emam et al., (2002b)	expert	“isoplaner/nonisoplaner image display”	monitor-based (vertical translation, pitch)	task completion time
				error rate
				leakage pressure
Wentink et al., (2000)	naive	“monitor position,” “camera rotation”	monitor-based (vertical translation, pitch)	task completion time
			camera-based (roll)	
			tool-based	
Rogers et al. (2012)	naive	“monitor position”	monitor-based (horizontal, vertical translation, pitch)	task completion time
Mandryk & MacKenzie, 1999	naive	“location of the image display”	monitor-based (vertical, horizontal translation, pitch)	task completion time
Inagaki et al. (2021)	naive, expert	“operator line of sight-monitor position”	“monitor-based (horizontal translation)	task completion time
				error rate
Miura et al. (2019)	expert	“monitor position,” “camera rotation angle”	monitor-based (horizontal translation) camera-based (yaw, roll)	task completion time
Cresswell et al., (1999)	expert	“camera rotation”	camera-based (roll, pitch)	task completion time
		“reverse alignment”		
		“camera alignment”		
Conrad et al. 2006	intermediate, expert	“rotational angle”	camera-based (roll)	task completion time
Gould & Frydman, 2007	intermediate, expert	“reverse-alignment”	camera-based (roll)	task completion time
Gallagher et al. (2009)	naive, expert	“camera rotation”	camera-based (roll)	task error rate
				task error rate
Hewitson et al., (2021)	naive, expert	“camera rotation”	camera-based (roll)	task completion time
				movement smoothness
				velocity
Emam et al., (2002a)	expert	“optical axis manipulations”	camera-based (yaw)	task completion time
				error rate
				leakage pressure
Ames et al., (2006)	naive, intermediate, expert	“camera angle”	camera-based (yaw)	task completion time
Rhee et al., (2014)	intermediate, expert	“viewing angle	camera-based (yaw)	task completion time
				task completion percentage,
				error rate
Dunnican et al., (2010)	naive, intermediate	“reverse alignment”	camera-based (yaw)	task completion time error rate
Wheeler et al., (2012)	naive	“camera rotation”	camera-based (yaw)	movement accuracy
Klein et al., (2015)	naive	“camera rotation”	camera-based (yaw)	task completion time
Neilson and Klein, 2018	naive	“camera rotation”	camera-based (yaw)	task completion time
				error rate (drops/completed transfer ratio)
Delicia and Griswold, 2011	naive	“viewing angle”	camera-based (pitch, yaw)	task completion time
				task completion percentage
Zhang and Cao (2012)	naive	“camera rotation”	camera-based (pitch, yaw, roll)	task completion
				error rate
Gallagher et al., (1998)	naive	“fulcrum effect	tool-based	error rate
Crothers et al. (1999)	expert	“fulcrum effect”	tool-based	error rate
Emam et al., (2000)	expert	“instrument length ratio”	tool-based	task completion time
White et al., (2014)	naive	“port location”	tool-based	movement smoothness
				movement smoothness

Columns (1) Study. (2) Cohorts. (3) Independent variables described within the study. (4) Perturbation types categorized according to our taxonomy. (5) Dependent variables described within the study. Note that the originally stated independent variables reliably capture whether the perturbation is monitor-, camera-, or tool-based, but fail to capture the specific identity of the perturbation applied (e.g., roll, pitch, yaw) that is afforded by our proposed nomenclature.

## Monitor-Based Sensorimotor Perturbations

The motor learning literature provides strong experimental evidence that monitor-based perturbations impose costs on motor planning and subsequent performance. Numerous studies have assessed human motor performance when the monitor is horizontally and/or vertically aligned with the physical workspace compared to when it is misaligned (Figure 2(b)). The consistent finding is that movements are less accurate and more variable when misaligned visual displays are used (Batmaz et al., 2017; Bédard & Proteau, 2005; Bo et al., 2006; Messier & Kalaska, 1997; Veilleux & Proteau, 2011). In general, movement control under image guidance appears to be more challenging than under direct viewing conditions (Batmaz et al., 2016, 2016b, 2017).

Several explanations have been proposed for these observations. First, under this type of perturbation, vision and proprioception no longer provide congruent information about hand position, making motor planning more difficult as both visual and proprioceptive information are typically used to plan reaches (Rossetti et al., 1995; van Beers et al., 1996; Van Beers et al., 1999). Second, when horizontal movements are represented vertically on a computer screen, additional sensorimotor transformations are required during motor planning (Ghez et al., 2000; Messier & Kalaska, 1997), and sensorimotor transformations are known to introduce noise and bias into motor planning (Churchland et al., 2006; McIntyre et al., 2000; Schlicht & Schrater, 2007; Sober & Sabes, 2003, 2005; Soechting & Flanders, 1989). In summary, the motor control literature strongly predicts that monitor-based perturbations should degrade laparoscopic performance and that aligning the visual display and the physical workspace (which approximates the conditions experienced in open surgery and routine visually-guided motor behavior) should benefit performance.

In the MIS literature, a substantial number of studies report improved task performance when the monitor is horizontally aligned with the workspace compared to when it is positioned to the left or right (Erfanian et al., 2003; Hanna et al., 1998; Haveran et al., 2007; Hernandez et al., 2014; Matern et al., 2005). Similar effects on performance have been observed when vertical monitor alignment is tested, with longer task completion times and/or more errors when the monitor is positioned at eye level compared to hand level (Hanna et al., 1998; Omar et al., 2005; Rogers et al., 2012). The performance benefits of vertical monitor alignment become more pronounced as task difficulty increases (Omar et al., 2005). There is an interesting tension here with the recommendations flowing from ergonomic considerations, where the preferred monitor height for reducing fatigue and neck discomfort is generally thought to be just below eye level.

The same general pattern of results is also evident in studies employing different experimental designs. For example, in one study (White et al., 2016), total task time was still shorter in the straight ahead monitor condition compared to horizontally displaced conditions. In another study (Emam et al., 2002b), vertical misalignment resulted in increased task execution time and errors compared to the aligned condition.

One outlier to this general trend is the classic study by Wentink et al. (2000), who reported significantly shorter task completion times in an eye-level condition compared to a workspace-level condition (Wentink et al., 2000). This discordant finding may be attributed to the unique design of the study, including the inclusion of camera rotation conditions and differences in monitor pitch angle across the tested conditions (for visualization of monitor pitch angle changes, see Figure 1(b)).

One general issue with many of the reviewed studies is that monitor pitch angle is often an uncontrolled variable. Changes in monitor pitch angle can introduce computationally demanding sensorimotor transformations, which can impact performance. However, the specific effects of monitor pitch angle changes are generally not reported in these studies.

Some studies manipulated monitor position but did not directly compare performance between different monitor positions (Inagaki et al., 2021; Mandryk & MacKenzie, 1999), or they manipulated various other elements concurrently with changes in monitor position (Miura et al., 2019), making the results difficult to interpret and compare with other studies of monitor-based perturbations.

In summary, monitor-based perturbations, which introduce misalignments between the monitor and the workspace, consistently impair motor performance in laparoscopic tasks, consistent with findings from the motor learning literature. The broad pattern of results suggests that placing the monitor at the same level and pitch as the surgical workspace could be a fruitful strategy for improving MIS performance by minimizing costly sensorimotor transformations required by the brain.

## Camera-Based Sensorimotor Perturbations

Various studies have investigated MIS task performance under visuomotor perturbations arising from rotations and/or translations of the laparoscopic camera (camera-based perturbations) (Figure 1(c)). These studies can be grouped according to whether they manipulate camera roll, yaw, or pitch. Roll describes rotation around the front-to-back axis of the camera (Figure 3(a), and (b)), yaw describes rotation around the side-to-side axis of camera (Figure 3(f), and (g)), and pitch describes rotation along the vertical axis of the camera (Figure 3(k), and (l)). Despite these being importantly different physical transformations with different effects on sensorimotor mappings, the literature often conflates them.

There are several other complicating factors for surgeon and camera operator performance that, for reasons of space, will not explored further here. For example, laparoscopes are typically available in straight ahead (90° scopes) and 30° (periscopic) orientations, with the latter introducing a combined yaw and roll under some circumstances. Moreover, operators can shift between scopes during a procedure. Finally, the camera is sometimes moved between access ports to facilitate a more advantageous viewpoint. Importantly, these changes may also increase cognitive load or otherwise alter the demands placed on motor control.

In many respects, camera-based perturbations are analogous to the visuomotor perturbations commonly employed in motor learning research (Krakauer, 2009; Krakauer et al., 2019). In these experiments, a novel mapping is imposed between the motion of a body part (e.g., hand) and the corresponding visual feedback provided about that body part, typically an on-screen visual cursor. The most commonly used visuomotor perturbation is a visuomotor rotation, where cursor feedback is rotated by some fixed angle relative to the direction of hand motion.

The general finding in motor learning research is that visuomotor rotations initially impair reaching performance, but participants gradually adapt and regain normal reaching abilities when error feedback is provided (Cunningham, 1989; Hay & Pick Jr, 1966; Held & Hein, 1958, 1963; Kohler, 1955; Krakauer et al., 2000, 2019; Redding et al., 2005; Welch, 1986). However, the specific time course of learning is often not reported in the MIS literature, except for a few exceptions (White et al., 2016). Thus, there is a significant gap in the MIS literature regarding the temporal aspects of learning and adaptation. Nevertheless, findings in the MIS literature align with the general pattern of impairment observed early in training, with performance in various laparoscopic tasks being degraded by camera-based perturbations, including roll and yaw, but not pitch. This basic pattern is also evident in the human factors literature (Ramesh et al., 2024).

The motor learning perspective also suggests that changes in pitch may not equally impair performance. Different perturbations induce different types of errors, which may be handled differently by the human motor system. Changes in camera yaw and roll induce directional movement errors, while changes in pitch introduce errors in movement extent (e.g., undershooting or overshooting). The motor learning literature indicates that adapting to errors in movement extent is, in some respects, easier than adapting to errors in movement direction (DeLucia & Griswold, 2011; Hanna et al., 1998; Krakauer et al., 2000; Pine et al., 1996).

The findings reported in the relevant MIS literature are generally consistent with the overall pattern observed in the motor learning literature: laparoscopic performance deteriorates when camera-based visuomotor perturbations are introduced and tends to worsen as the size of the perturbation increases. However, this pattern only holds for manipulations of camera roll and not yaw or pitch.

### Camera Roll

Studies have consistently reported degraded task performance when the camera view is misaligned relative to the physical workspace, particularly when the camera roll angle is greater than 0° (Conrad et al., 2006; Cresswell et al., 1999; Gallagher et al., 2009; Gould & Frydman, 2007; Hewitson et al., 2021; Wentink et al., 2000). Reverse alignment, where the camera is positioned 180° opposite to the surgeon, has been of particular interest due to its potential to reduce the performance costs associated with the fulcrum effect. However, not only inexperienced residents (Gould & Frydman, 2007) but even expert laparoscopic surgeons exhibit increased task errors and completion times under reverse alignment viewing conditions compared to normal forward alignment (Cresswell et al., 1999). Laparoscopic training can partially mitigate the negative effects of reverse alignment, but performance remains worse compared to aligned viewing conditions (Crothers et al., 1999).

Performance also deteriorates with increasing camera roll angles other than 180°. Both surgical residents and experienced surgeons show linearly increasing task completion time and errors as camera roll angle increases (Conrad et al., 2006). Laparoscopic expertise can help overcome the negative effects of camera roll-based perturbations in certain task contexts, as seen in the performance of surgeons compared to residents in knot-tying tasks (Conrad et al., 2006). However, the reasons for these contextual differences are not well understood. Similar findings are observed when manipulating the orientation of the task workspace instead of the camera view (Emam et al., 2002b). Horizontal tasks displayed vertically and vice versa lead to degraded laparoscopic performance. Finally, a recent study investigated the impact of camera realignment to a 0° camera roll angle on laparoscopic performance (Hewitson et al., 2021). The results showed that camera realignment did not improve performance but instead consistently degraded performance compared to the unrotated conditions across both novice and expert participants. Although it goes beyond the scope of the current review, it is worth acknowledging that researchers investigating the effects of camera viewpoint changes in teleoperation also report very similar results (Almeida et al., 2020; DeJong et al., 2011; DeJong et al., 2004; Hiatt & Simmons, 2006; Macedo et al., 1998).

### Camera Yaw

Several studies have investigated the effects of camera yaw angle on laparoscopic task performance (Ames et al., 2006; DeLucia & Griswold, 2011; Emam et al., 2002a; Haveran et al., 2007; Klein et al., 2015; Rhee et al., 2014; Wheeler et al., 2012). When the camera view was displaced to the left or right, both expert surgeons and novice participants showed a decline in performance (Haveran et al., 2007). A departure from this general pattern was observed in one study where task errors and execution times increased when the camera was positioned on the right side but remained unchanged when positioned on the left (Emam et al., 2002a). This result could simply reflect the small sample size used in the study (*n* = 10), or the left-right asymmetry could be real but reflect idiosyncratic task features. For example, it may be the case that in this particular task—a laparoscopic suturing task in which the dominant hand is likely to play a primary role—the camera positioned on the left (nondominant) side may offer a more favorable viewpoint compared to the right (dominant) side. Many of the other studies reviewed above used a bimanual peg transfer task, which places more equal demands on both hands. So perhaps this explains the discrepancy. Future studies will be needed to resolve this issue.

Similar findings were reported in studies with different numbers of camera yaw angles tested (Ames et al., 2006; DeLucia & Griswold, 2011; Zhang & Cao, 2012). When one yaw angle manipulation (90°) was compared to a control condition (0° yaw angle), slower movement completion times were observed (DeLucia & Griswold, 2011). When five different yaw angles were tested (0–180°), task completion time and errors tended to increase with increasing camera yaw angles for novice, intermediate, and expert surgical groups (Ames et al., 2006; Rhee et al., 2014). Dunnican et al. (2010) also found that training under misalignment conditions conferred performance benefits (i.e., ‘positive generalization’, or ‘transfer’ of performance) across both aligned and misaligned conditions, underscoring the importance of exposure to different yaw angles during training (Dunnican et al., 2010). A recent study aimed to investigate the extent of these generalization effects (Tharp et al., 2023). Under the assumption that an axis inversion between yaw angles would facilitate skill transfer, participants were trained in a laparoscopic pointing task under a 45° camera yaw and then tested in the same task under a 135° yaw angle. Contrary to their expectation, training under a 45° yaw did not generalize to the 135° condition, suggesting that participants could not utilize axis inversion directly. This finding underscores how generalization of learning from one direction to another might be complex and highly direction-dependent.

Several studies involving the rotation of the entire apparatus along the yaw axis also showed that increasing camera yaw angles led to degraded laparoscopic performance, with performance costs peaking at 135° and then showing a relative improvement at 180° (Klein et al., 2015; Neilson & Klein, 2018; Wheeler et al., 2012). Overall, these results contribute to our understanding of the sensorimotor challenges posed by different camera yaw angles in laparoscopic surgery.

### Camera Pitch

The influence of camera pitch, specifically the optical axis-to-target view angle (OATVA), on laparoscopic task performance has received less attention compared to camera roll and yaw. Changes in camera pitch have ergonomic implications but do not significantly alter the relationship between hand and tool-tip movement, making their impact on surgical performance less significant. In a foundational study by Cresswell et al. (1999), no significant differences in task performance were observed across three different camera pitch angles (45°, 60°, 90°) (Cresswell et al., 1999). However, several studies have reported better performance in overhead viewing conditions where a relatively large pitch angle or OATVA was used compared to smaller pitch angles aligned with the surgeon’s viewpoint (DeLucia & Griswold, 2011; Hanna & Cuschieri, 1999; Zhang & Cao, 2012).

To summarize, motor performance in various laparoscopic tasks is affected by camera-based sensorimotor perturbations. The costs incurred are proportional to increasing deviations of the laparoscopic camera along the roll and yaw axes relative to the workspace or surgeon-aligned view. Expert laparoscopic surgeons generally experience less impairment than novices under these manipulations. It is important to differentiate between different camera-based manipulations in the experimental literature, as the effects of perturbations induced by changes in camera pitch differ from those induced by roll and yaw adjustments.

Similar findings have been reported in the motor control literature, where visuomotor rotations initially hinder reaching performance but participants gradually adapt and regain normal performance levels (Cunningham, 1989; Hay & Pick Jr, 1966; Held & Hein, 1958, 1963; Kohler, 1955; Krakauer et al., 2000, 2019; Redding et al., 2005; Welch, 1986). However, the specific time course of learning is often not reported in the minimally invasive surgery (MIS) literature, with few exceptions such as the study by White et al. (2016). Enhancing MIS training could involve incorporating systematic manipulations of camera roll and yaw, particularly with larger angles, to facilitate skill acquisition.

## Tool-Based Sensorimotor Perturbations

Humans are remarkably adept at using a wide variety of tools. Yet learning to dexterously manipulate tools, including laparoscopic instruments such as a needle driver, presents serious challenges. Specifically, we must learn the novel input–output properties of the controlled object (Heuer & Sülzenbrück, 2009, 2013). The neural mechanisms supporting this form of learning, known as *de novo learning* (Krakauer et al., 2019), has attracted increasing attention among motor-learning researchers (Flanagan & Wing, 1997; Heald et al., 2018; Imamizu, 2010; Imamizu et al., 2000; Imamizu & Kawato, 2012; Mehta & Schaal, 2002). This presents another promising opportunity for MIS researchers to leverage findings from the field of motor learning. Consider the process of learning to use a new hand-held tool. First, it requires acquiring a new kinematic transformation, which is the mapping of hand movements (grasping the tool handle) to movements of the tool tip (e.g., the fulcrum effect). Even for relatively simple tools like first-order levers, which share similarities with laparoscopic tools, learning this inverted mapping between the movement direction of the tool tip and that of the hand is necessary (Heuer & Sülzenbrück, 2013).

Researchers have observed that when hand movements are transformed into inverted movements of a tool, movement initiation time and error rates increase, indicating higher demands on motor planning (Kunde et al., 2007; Müsseler et al., 2008; Müsseler & Skottke, 2011). Comparable findings have been observed in more applied settings such as in the control of mining equipment (Burgess-Limerick et al., 2010), where operators have to learn an inverted mapping between the control lever and movements of the controlled object (e.g., boom or drill bit) compared to a direct mapping. The effects of extensive experience with tools have also been investigated. Experts who have mastered the control of tools with complex kinematic transformations (such as inversions) prioritize the desired distal action effects over the proximal action effects (Kunde et al., 2007; Massen, 2013; Massen & Prinz, 2007; Müsseler & Skottke, 2011; Sutter et al., 2011, 2013). In other words, experts learn to focus on controlling the distal tool tip while ignoring the proximal control of the hand.

Another critical aspect of learning to use a tool like a first-order lever is learning to adjust to and control its gain. Gain refers to the relationship between the amplitude of hand movement and the resulting movement at the lever tip. Laparoscopic instruments can be formally characterized as first-order levers with a variable fulcrum (Heuer & Sülzenbrück, 2009). As the fulcrum point changes, the length of the so-called load arm (the effective, distal portion of the lever terminating in the instrument tip) and effort arm (the proximal portion of the lever terminating in the handle) vary. Gain will depend on the type of movement being performed and the current fulcrum point of the tool (Heuer & Sülzenbrück, 2009, 2013). Changes in visuomotor gain, such as the relationship between the distance moved by the hand and the distance moved on the screen by a visual cursor representing the hand, is known to alter motor performance (Bohan et al., 2010; Fernandez & Bootsma, 2004). It also induces motor adaptation, but it looks different from the adaptation that results from the application of visuomotor rotations—specifically adaptation tends to occur more rapidly and generalization of learning to novel contexts is greater (Krakauer et al., 2000; Pine et al., 1996; Vindras & Viviani, 2002; Wei et al., 2014). Experimental evidence suggests that different brain mechanisms support the learning of visuomotor rotations compared to learning inversions or mirror-reversals, even though a 180-degree rotation is mathematically equivalent to an inversion (Telgen et al., 2014). This suggests that training to cope with tool-based perturbations like the fulcrum effect could be conducted independently of training for camera- or monitor-based perturbations.

Although outside the focus of this review, the use of tools introduce other complicating factors for sensorimotor control including the need to compensate for subtle variations in the mass and inertia of hand-held tools can influence task performance (Hoffmann et al., 2022; Lin et al., 2011; Silva et al., 2016). Additionally, different instrument tips have different jaw lengths and mechanisms of action that must be learned (e.g., some tips have symmetrically opening jaws while for others only one jaw moves). Finally, switching between instruments during a procedure likely imposes other demands on sensorimotor control.

In summary, the motor learning perspective predicts that novel tool use in MIS initially leads to performance costs, which gradually decrease as the input–output mappings (i.e., internal model) of the new tool are acquired. Even after developing a high-quality internal model, regular recalibration is necessary to cope with the sensorimotor perturbations commonly encountered in MIS, resulting in effects similar to those discussed earlier regarding monitor- and camera-based perturbations.

Several pioneering studies have investigated tool-based perturbations in MIS. In one study (Gallagher et al., 1998), surgically novice participants performing a laparoscopic cutting task under different types of visual feedback. They found that participants who received *y*-axis inverted visual feedback, effectively eliminating the fulcrum effect, made significantly more correct incisions compared to those who received unaltered visual feedback, indicating the influence of the fulcrum effect under normal laparoscopic conditions. In a related study, Crothers et al. (1999), tested the effects of *y*-axis image inversion on experienced laparoscopic surgeons. Discussed in the previous section as an instance of camera roll manipulation, this study also relates to tool-based perturbations as the roll manipulations aimed to compensate for the visual feedback changes induced by the fulcrum effect. Interestingly, image inversion improved performance among novices, but not for experienced surgeons who showed better performance under normal laparoscopic viewing conditions, indicating that experienced surgeons have learned to adapt their responses to the fulcrum effect.

Another study (Emam et al., 2000) investigated the effects of movement gain in a laparoscopic knot tying task. Surgeons took significantly longer to complete the task under the high-gain condition, where the load arm of the instrument was twice as long as the effort arm, compared to the other two conditions with lower gain. A final study (White et al., 2014) investigated the benefits of training across multiple instrument port sites in a laparoscopic task. Novice participants were divided into two training groups, with one group training across multiple port locations representing different tool-based perturbations. The study showed that the multiple-port training group outperformed the single-port training group when tested on a new port location they had not encountered during training, indicating the potential benefits of incorporating multiple tool-based perturbations into MIS training.

Despite the limited number of studies in this area, the general finding is that tool-based perturbations tend to degrade performance unless individuals have extensive experience controlling these tools.

## Discussion

It’s important to note that the majority of the studies reviewed were performed in simulated environments, which cannot fully capture the sensorimotor complexity and stress factors inherent in a real surgical context (Spiliotis et al., 2020; Tjønnås et al., 2022; Weersink et al., 2019). Naturally, future research must continue to explore ways to bridge the gap between simulated and real surgical contexts. Nonetheless, our review aims to taxonimize the typical range of sensorimotor distortions that affect MIS skill learning, rather than capturing all complex sensorimotor perturbations that exist within a real surgical context. Despite the limitations of surgical simulation training, it remains a valuable tool for understanding the sensorimotor distortions that affect MIS skill learning. 

A number of important insights emerge from this review that can help accelerate progress in the field. The first insight to emerge is just how crucial it is for researchers to be explicit about which type of perturbation is being tested in their study. Many of the studies we reviewed here use terminology in an ambiguous way that conflates importantly different types of sensorimotor perturbations, which should be kept distinct. The taxonomy we have proposed brings much needed clarity and structure to the existing literature, revealing key similarities and differences between different experimental studies. This taxonomy provides a crucial first step towards more informative cross-study comparisons and enhancing the reproducibility of research in the field (Ioannidis, 2005; National Academies of Sciences, Medicine, et al., 2019).

Another important insight is that the existing literature tends to focus on macroscopic performance metrics such as error rates and overall task completion time, the latter of which may be especially difficult to relate to real surgical contexts such as tissue dissection where slower movements emphasizing accuracy over speed (Fitts, 1954; MacKenzie, 1992, 2018) are required to cleanly separate tissue along an anatomical plane (Patkin, 2008).

Alternative, finer-grained kinematic metrics including instrument path length, velocity, acceleration, and smoothness/jerk, may provide more appropriate task-relevant measures, whose appropriateness will change depending on the specific surgical context of interest (Aghazadeh et al., 2023; Culmer et al., 2009; Flash & Hogan, 1985; Hogan & Sternad, 2009; Huegel et al., 2009). Another benefit of these metrics over gross movement time is that they are sensitive not only to widespread speed-accuracy trade-offs, but also to the different sub-components of voluntary movements: the largely ballistic primary component toward the target and the secondary corrective component guided by visual feedback (Meyer et al., 2018; Woodworth, 1899).

As one example, consider the limitations of relying on task completion time to investigate the impact of gain on laparoscopic performance. Since high-gain scaling allows for rapid initial movement but slower final acquisition, while low gain results in the reverse (Akamatsu & MacKenzie, 2002; Slocum et al., 2005; Thompson et al., 2007), using a metric insensitive to these different components of the movement may obscure real patterns present in the raw data. The adoption of more fine-grained metrics in future research could therefore reveal interesting aspects of surgeon behavior that might otherwise remain hidden.

Clearer terminology alone does not equip researchers with all the necessary tools to generate more precise predictions capable of guiding future experiments. This is the role for general theory (Muthukrishna & Henrich, 2019). As we have now seen, a critical problem with the reviewed literature is that it is often extremely challenging to relate findings from one study to another. In the absence of a general theory, all that can be inferred safely from a single experiment is that the observed results hold for a specific group of participants in a specific experimental context. Unfortunately, this situation describes the current state within much of the MIS literature, compounding the difficulties associated with imprecise terminology. A general theory would help to unify disparate studies and provide an overarching framework for relating them. Drawing on the computational motor learning perspective serves as a valuable starting point in achieving this crucial goal.

As we have already seen, the computational motor learning perspective (Shadmehr, Criscimagna-Hemminger et al., 2010; Shadmehr & Krakauer, 2008; Shadmehr & Wise, 2005; Wolpert & Ghahramani, 2000; Wolpert et al., 2011) provides a wealth of valuable insights into how we select, plan, execute, and adapt our movements in response to perturbations in the external environment including exactly the sort encountered during MIS. Several key principles stand out as most relevant to the literature covered in this review.

First, movement planning and execution in MIS contexts are highly nontrivial from the point of view of the central nervous system. In general, movement planning requires *sensorimotor transformations*, which involve converting sensory signals into motor commands (Pouget & Snyder, 2000). Even to perform a simple action such as reaching for a glass of water on the table, the brain must determine the precise set of muscle activations and joint rotations to bring the hand to the target. Since information about target location is initially specified in visual coordinates, sensorimotor transformations are required to make that information usable by brain regions involved in generating the appropriate motor commands. Importantly, the monitors, cameras, and tools ubiquitous in MIS necessitate additional sensorimotor transformations on top of those required for ordinary visually-guided movements, and therefore further complicate the motor control problem the central nervous system must solve.

The second insight is that motor performance is rarely if ever stationary. This is true for both the environment in which motor behavior occurs (e.g., wind conditions on a golf course or camera rotations during laparoscopic surgery) and properties of the motor system itself (e.g., fatigue of the relevant muscles) are subject to continual change. These sensorimotor perturbations necessitate continual tuning of the mapping between motor commands and sensory feedback, which is critical for the maintenance of consistent performance in a fluctuating environment (Krakauer et al., 2019; Shadmehr, Smith, et al., 2010; Wolpert et al., 2011). Researchers aiming to comprehend the contours of laparoscopic performance would benefit from considering the significant role of motor adaptation (Crossley et al., 2021).

The third insight is that very few studies actually measure motor learning—that is, how performance improves over time (see Crothers et al., 1999, Dunnican et al., 2010; White et al., 2016, for exceptions). Instead, focus tends to be on the short-term impact of visuomotor perturbations on performance. In our estimation, this neglect of learning is probably the biggest limitation of the literature to date. Although the majority of studies reviewed assess mean task performance averaged over an entire experimental session, it is unlikely that individual behavior is stationary over this period. It would therefore be informative to explore and quantify how performance changes as participants learn the experimental task. Given that learning MIS can be prohibitively difficult such that some trainees never reach proficiency (Buckley et al., 2014; Green & Bavelier, 2003), understanding precisely how sensorimotor perturbations influence learning in MIS contexts could inform how surgeons are trained.

The computational motor learning perspective (Shadmehr, Criscimagna-Hemminger et al., 2010; Shadmehr & Krakauer, 2008; Shadmehr & Wise, 2005; Wolpert & Ghahramani, 2000; Wolpert et al., 2011) can help provide a deeper scientific understanding of learning in MIS. By drawing on sophisticated behavioral paradigms and computational modeling techniques common to this scientific domain, researchers can begin to decipher different aspects of motor learning in MIS including savings and generalization (see, e.g., Hewitson et al., 2020). For example, using these resources one can quantitatively test whether savings, defined as faster performance gains during a second exposure to a perturbation (Avraham et al., 2021; Krakauer et al., 2005), might be differentially expressed in expert surgeons who must frequently switch between several task contexts with rather different task demands (e.g., between laparoscopic and robotic or open surgery).

Another critical question at the center of contemporary motor neuroscience concerns the interplay between low-level motor adaptation—an implicit, automatic process—and higher level, conscious compensatory strategies in response to environmental changes that require behavioral adjustments (Albert et al., 2022; McDougle et al., 2015). A host of innovative experimental methods and models are actively being developed to decipher the relative contributions of implicit versus explicit processes in motor learning. This presents an exciting opportunity for researchers interested in MIS because the role that each of these learning processes might play in surgical contexts remains entirely unknown. Exploring what reflective strategies expert laparoscopic surgeons might flexibly use to boost their performance and compensate for environmental perturbations could in turn lead to valuable insights that accelerate learning for new trainees (Gilmore et al., 2022).

## Conclusion

In this review, we have proposed a standardized taxonomy to help classify the fragmented literature on sensorimotor learning and performance in minimally invasive surgery. We have argued that the motor learning perspective offers a powerful, unifying framework to help put the field on a more solid theoretical footing. It is our hope that this literature review, and the recommendations we have provided, will help to promote further gains in our scientific understanding of the factors influencing sensorimotor learning and performance in MIS. While this is an important goal in its own right, our greater hope is that this deeper understanding will catalyze changes in surgical training, ultimately leading to improved patient outcomes. Our contribution is only a small first step in this direction.

## Key Points

• **Sensorimotor challenges in MIS:** Although many factors contribute to the difficulties encountered in MIS, sensorimotor factors likely have a large and underappreciated impact on laparoscopic performance.
• **Clarifying the experimental literature:** We propose a standardized terminology and precise taxonomy for describing experimental manipulations used in MIS research that will significantly reduce fragmentation in the literature, if widely adopted.
• **The value of the computational motor learning perspective:** The computational motor learning perspective can help to deepen our scientific understanding and facilitate discovery of the principles underlying learning and performance in MIS.
